# A novel role of BPCs in the control of medial domain differentiation during gynoecium development in *Arabidopsis thaliana*

**DOI:** 10.1007/s11103-025-01662-x

**Published:** 2025-12-23

**Authors:** Francesca Caselli, Micaela Palermiti, Rosanna Petrella, Veronica Astrid Morlacchi, Kai Dünser, Jűrgen Kleine-Vehn, Matteo Chiara, Veronica Gregis

**Affiliations:** 1 Dipartimento di Bioscienze, Università degli Studi di Milano, Milan, Italy; 2 Institute of Biology II, Chair of Molecular Plant Physiology (MoPP), University of Freiburg, 79104 Freiburg, Germany; 3 Center for Integrative Biological Signalling Studies (CIBSS), University of Freiburg, 79104 Freiburg, Germany; 4 Institute of Molecular Plant Biology (IMPB), University of Natural Resources and Life Sciences (BOKU), Vienna, 1190 Vienna, Austria

**Keywords:** Basic pentacysteine (BPC), SPATULA (SPT), Septum, Carpel margin meristem (CMM), Gynoecium development

## Abstract

The gynoecium, a highly specialized structure in flowering plants, ensures their high reproductive success through the control of different crucial steps spanning from ovule protection to fertilization and seed maturation and dispersion. Multiple *bpc* mutants show reduced vigor, small fruit size and height, a reduced number of seeds and problems in septum fusion and formation. BPCs are known to be involved in the regulation of key factors involved in plant development, and they are thought to function both as activators and repressors of target gene expression. Here we showed that gynoecium development is affected in different multiple mutants of the *Basic PentaCysteine* (*BPC*) genes, where the septum fails to develop properly, and that BPCs of class I and II regulate the expression of different genes involved in carpel development and phytohormonal pathways regulation. Considering the fundamental role of the gynoecium, which affects the reproductive success of the plants, we focused on understanding which genes could be putative direct targets of BPCs and thus involved in gynoecium development. We demonstrated that *SPATULA* and *NO TRANSMITTING TRACT* (*NTT*), which play pivotal roles in carpel and transmitting tract development, are downregulated. As a consequence, *bpc* multiple mutants fail to properly develop the septum and the transmitting tract. Interestingly, among the downregulated genes, we also found *PIN-LIKES3*, whose promoter can be directly bound by BPCs, which is an auxin efflux carrier that regulates and controls cytoplasmic availability of auxin and could also contribute to various growth processes.

**Supplementary Information:**

The online version contains supplementary material available at 10.1007/s11103-025-01662-x.

## Key message

BPC transcription factors orchestrate carpel formation by regulating key targets—including SPATULA and NTT—highlighting their direct, essential role in gynoecium and septum development.

**Supplementary Information:**

The online version contains supplementary material available at 10.1007/s11103-025-01662-x.

## Introduction

The gynoecium is one of the most complex organs produced by angiosperms and confers several evolutionary advantages to flowering plants, ensuring a high rate of reproductive success. The gynoecium not only encloses and protects the ovules, but it also functions to capture pollen grains and guide the germinating pollen tubes to the ovules. Furthermore, once fertilization has occurred, the carpel acquires the identity of fruit, harboring the seeds and allowing their dispersion upon maturation (Ferrándiz et al. 2010).

In Arabidopsis, the gynoecium is a bilocular hollow tube, formed by two congenitally fused carpels. This structure starts to arise from the center of the flower at stage 5. Later, carpel walls will differentiate into the valves, while the margins of the fused carpels will start to proliferate inside the hollow tube, giving rise to the medial domain. At stage 10, the two proliferating medial domains fuse in the middle of the gynoecium, leading to the formation of a bilocular structure (Sessions 1997). The medial domain is of fundamental importance, as within this tissue the Carpel Margin Meristem (CMM) differentiates. This meristematic tissue will later give rise to all the medial organs and tissues, among which are ovules, placenta, septum, and transmitting tract (Reyes-Olalde et al. 2013).

Here we showed that BASIC PENTACYSTEIN proteins play a key role in orchestrating the development of this fundamental structure, not only by controlling the expression of several pivotal regulators of gynoecium development, such as *SPATULA* and *NO TRANSMITTING TRACT*, but also through the direct regulation of the auxin carrier *PIN-LIKES 3* within the carpel margin meristem.

BPCs are plant-specific transcription factors, which can act both as activators and repressors of transcription by binding to specific “GA”-rich sequences. In Arabidopsis, this family is composed of seven members, divided into three classes: class I, which includes *BPC1 BPC2*, and *BPC3*, class II, containing *BPC4 BPC5*, and *BPC6* and class III, whose only member is *BPC7.* These factors, with the exception of *BPC5*, which is a pseudogene, are broadly expressed throughout Arabidopsis vegetative and reproductive growth (Meister et al. 2004; Monfared et al. 2011).

Single and double *BPCs* mutants have a wild type-like phenotype; however, the analysis of higher-order mutants allowed a better understanding of the redundancy among the genes of this family. Indeed, mutating all the BPCs of class I (*bpc1*,* bpc2*, and *bpc3*) leads to the onset of severe developmental defects in the inflorescence (Monfared et al. 2011; Simonini and Kater 2014). The mutation of the BPCs of class II, on the other hand, does not cause any visible phenotype (Monfared et al. 2011). Interestingly, in the quintuple mutant, lacking both BPCs of class I and II, the phenotypical defects are enhanced compared to the ones of the *bpc1 bpc2 bpc3* mutant, suggesting a genetic interaction between these two classes of genes (Petrella et al. 2020). Finally, the mutation of *BPC7* alone and in combination with other higher-order mutants does not show or add any phenotypical defect (Monfared et al. 2011).

BPCs exert their function as transcriptional regulators by interacting among themselves and with other factors. One of the better characterized molecular mechanisms of action of the BPCs is indeed their role in the negative regulation of the homeotic gene *SEEDSTICK (STK)*. Both BPC of class I and II can interact with SVP forming a complex that directly binds the promoter of *STK*, inducing DNA looping in this region. The subsequent repression of *STK* is achieved through the recruitment of the Polycomb Repressive Complex 1 (PRC1), which deposits repressive histone markers to induce silencing of the target genes (Kooiker et al. 2005; Simonini et al. 2012; Hecker et al. 2015; Petrella et al. 2020). Similarly, BPCs of class I can interact with members of the PRC2, to correctly confine the expression of *FUSCA3* during seed development (Wu et al. 2020).

In Arabidopsis, BPCs are implied in several developmental processes, spanning from leaves morphology (Lee et al. 2022; Monfared et al. 2011) and stomata development (Liu et al. 2020; Samakovli et al. 2020) to ovules and seed/embryo maturation (Petrella et al. 2020; Wu et al. 2020). Additionally, BPCs were recently correlated with the perception of endogenous stimuli such as circadian oscillations, salinity, and osmotic tolerance (Lee et al. 2022; Li et al. 2021; Yan et al. 2021). Finally, BPCs also appear to be associated with signaling processes involving various hormones, including cytokinin and abscisic acid (Mu et al. 2017; Shanks et al. 2018; Simonini and Kater 2014) and auxin biosynthesis control (Zhao et al. 2024).

Even though our understanding of the functions of these widely expressed factors is continuously expanding, many of the pleiotropic phenotypical defects displayed by BPCs multiple mutants are still unexplained. With our work, we elucidated their role during the development of the female reproductive organ, the gynoecium.

## Materials and methods

### Plant material and growth conditions

*Arabidopsis thaliana* Columbia-0 ecotype (Col-0) was employed in all the experiments. Plants were grown in a controlled environment at 20–22 °C under long-day (16 h light/8 h dark) conditions. The *bpc1-2 bpc2 bpc3* (*bpc123*) and the *bpc1*-2 *bpc2 bpc3 bpc4 bpc6* quintuple mutant (*bpcV*) were previously obtained in our laboratory (Simonini and Kater 2014; Petrella et al. 2020). *bpc4 bpc6* were obtained from Monfared et al. 2011. *spt-11* insertional line corresponds to line WISCDSLOX466B7 from the WiscDsLox T-DNA collection in ABRC and was already described in Ichihashi et al. 2010. The *pBPC6:GFP-BPC6* line employed here was previously described in Shanks et al. 2018.

The *pils3 pils4* double mutant lines were generated using CRISPR/Cas9 gene editing technology (Wang et al. 2015). Mutants were identified in T2 by Sanger sequencing. Lines were back-crossed with Col-0, and homozygous lines were re-sequenced. The identified lines contained a 1 bp insertion in Exon 1 of both *PILS3* and *PILS4*, resulting in premature stop codons in both cases. gRNA and sequencing primers: PILS3 sgRNA target: GGATTTTATATGGCTCTCGATGG; PILS4 sgRNA target: AGCTTCATCGAAACCAGTTGTGG and fwd: TCTTCGGCGGTTCTTGCAGCTC; rev: CCGGCATAAACCACCTGCAATGC (Supplementary Fig. 1). All the mentioned published mutants were genotyped according to the cited literature. Primers employed for genotyping are listed in Supplementary Table 6.

### Technovit inclusion and slide staining

Whole Arabidopsis inflorescences were collected and fixed in FAA (50% ethanol, 5% acetic acid, 3.7% formaldehyde) for 15’ under vacuum and O/N at 4 °C. Gynoecia at the developmental stages of interest were manually dissected from the inflorescence and rehydrated in a series of ethanol solutions. To facilitate the correct orientation of the gynoecia, these are pre-embedded in 1.5% agarose and then dehydrated by passing through a series of ethanol solutions. The samples were then embedded in Technovit^®^ 7100 according to manufacturer instructions (Heraeus Kulzer, Germany). Technovit blocks were sectioned using a microtome (thick section of 10 μm), placed onto glass slides, and air dried.

The slides were stained with 0.5% Alcian blue 8GX (pH 3.1) for 25’ and 0.5% Neutral Red Solution for 5’. Slides were mounted with BioMount and analyzed with the aid of a Zeiss Axiophot D1 microscope equipped with differential interference contrast optics. Flower developmental stages were assigned according to (Smyth et al. 1990).

### RNA extraction and gene expression analysis

Total RNA was extracted from inflorescences using the LiCl method (Verwoerd et al. 1989). RNA samples were treated with DNase and retrotranscribed using the iScript™ gDNA Clear cDNA Synthesis Kit (BIO-RAD). Diluted cDNAs were used as a template for RT-PCR. Transcript detection was carried out with iQ Sybr Green Supermix 2X (Bio-Rad) in a Biorad C1000™ thermal cycler on three technical replicates. Three biological replicates for each experiment were performed. Arabidopsis reference gene UBIQUITIN (At4g36800) was used as an internal reference during the experiments. Employed primers are listed in Supplementary Table 6.

### In situ hybridization analysis

*Arabidopsis thaliana* inflorescences were fixed in FAA (ethanol 50%; acetic acid 5%; formaldehyde 37%), under mild vacuum conditions for 30’, dehydrated in a graded ethanol series, transferred to Bioclear (Bioptica) and then embedded in Paraplast X-tra^®^ (Sigma-Aldrich). Tissue Sect. (7 μm) were hybridized with digoxigenin-labeled RNA antisense or sense probes following the protocol published by Coen and Meyerowitz (1991). *SPT* and *NTT* probes were already described in Heisler et al. (2001) and Crawford et al. (2007), respectively. The newly designed *PILS3* probe specificity was validated by employing the sense probe to hybridize the same tissues employed for the antisense probe. The primers employed for probe amplification are listed in Supplementary Table 6. The sections were analyzed with the aid of a Zeiss Axiophot D1 microscope equipped with differential interference contrast optics. Flower developmental stages were assigned according to Smyth et al. (1990).

### RNA sequencing and DEGs analysis

Total RNA was extracted from 3 biological replicates from both wild type and *bpcV* mutant inflorescences apices with flowers up to stage 12 using NucleoSpin RNA Plant kit from Macherey Nagel. Sequencing libraries were prepared and sequenced on the HiSeq Illumina platform. Paired-end sequencing was performed. Reads were aligned to the Arabidopsis TAIR10 annotation of the *A. thaliana* transcriptome with bowtie2 (Langmead and Salzberg 2012). Gene expression levels were estimated by RSEM (B. Li and Dewey 2011) and differential analyses of gene expression were executed with edgeR (Robinson et al. 2010). The Genewise Negative Binomial Generalized (glmQLFTest) was applied to test for statistically significant differences. P-values were corrected using the Benjamini Hochberg procedure for the control of the False Discovery Rate and only genes showing a p-value < = 0.01 following the FDR adjustment were considered as differentially expressed (DEGs).

Functional enrichment analyses were performed using the ShinyGo 0.76 web interface on the complete collection of DEGs.

### Pscan and enrichment of TF families

Pscan (Zambelli et al. 2009) was employed to identify over-represented position frequency matrices (PFMs) in the promoters of DEGs. PFMs were obtained from the “non-redundant” core collection of plant PFMs as available from the 2020 release of the Jaspar database (https://jaspar.genereg.net/). Promoters were defined as regions spanning 1100 bp (from − 1000 upstream to + 100 bp downstream) from the transcription start site (TSS) of the TAIR 10 gene models. The Benjamini-Hochberg procedure for the control of the False Discovery Rate (FDR) was applied and PFMs, with a corrected pscan *p* value < 0.01 were considered significantly enriched.

Transcription factors (TFs) and corresponding transcription factor binding sites (TFBS) were assigned to a family based on the Jaspar 2020 annotation of Transcription factor families in plants. To identify families of TFs over-represented in any set of DEGs, a contingency table with the following data was computed for every TF family: (a) total number of TFs in the family significantly enriched according to pscan; (b) total number of TFs in the family; (c) total number of TFs of any family significantly enriched according to pscan; (d) total number of TF in Jaspar 2020. A Fisher exact test was applied to test for statistically significant over-representation. P-values were corrected by the Benjamini and Hochberg procedure for the control of False Discovery Rate.

### Chromatin ImmunoPrecipitation (ChIP)

Chromatin immunoprecipitation was performed on whole inflorescences, with flowers up to stage 13 – when anthesis occurs - as described in Gregis et al. (2009). GFP-Trap^®^Magnetic Agarose (Proteintek) was employed to immunoprecipitate the GFP-BPC6 protein. The enrichment of the regions of interest was measured through RT-PCR, as described above. Three biological replicates were performed for each experiment. Fold enrichment was calculated following Matias-Hernandez et al. 2010; normalizing the fold change on *ACTIN7.* Employed primers are listed in Supplementary Table 6.

## Results

### bpc multiple mutants display septum formation defects

The simultaneous mutation of all the BPCs of class I (*BPC1*,* BPC2*, and *BPC3*) causes pleiotropic developmental defects, both during their vegetative and reproductive growth. Such defects are exacerbated in plants in which also BPCs of class II (*BPC4* and *BPC6*) are mutated. These mutants are indeed characterized by reduced vigor and fitness, and a smaller fruit and leaf size (Monfared et al. 2011; Simonini and Kater 2014; Petrella et al. 2020).

**Fig. 1:**
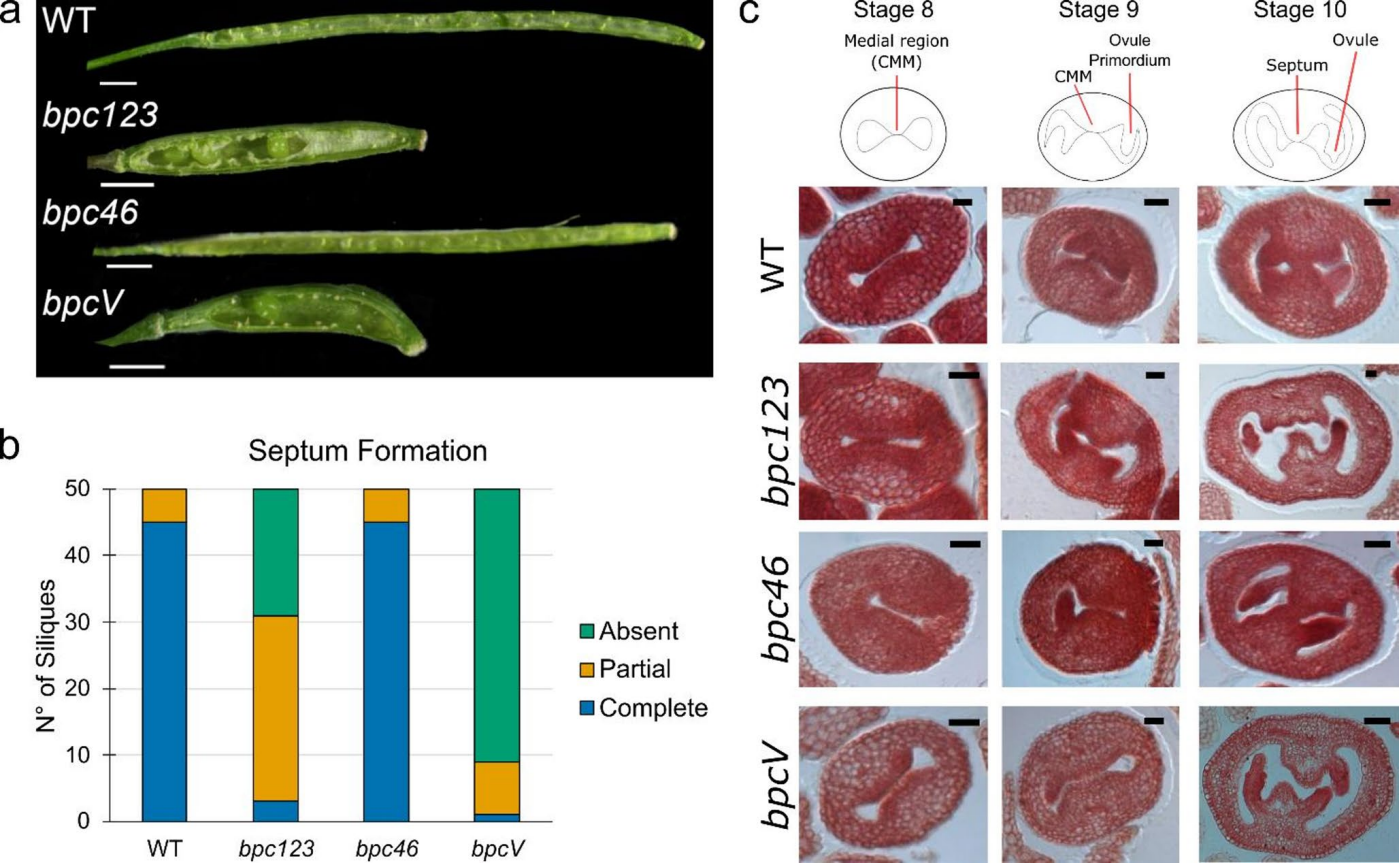
Characterization of multiple *bpc* mutants. **a** Stereomicroscope images comparing opened silique, from which one of the two valves and ovules were removed to show the septum, in wild type, *bpc123*, *bpc46*, and *bpcV* mutants. Scale bar 1 mm. **b** Stacked bar chart showing septum formation, divided into complete, partial or absent, in wild type, *bpc123*, *bpc4 bpc6*, and *bpcV*. 10 siliques were analyzed from 5 independent plants for a total of 50 siliques for each genotype. The chart displays the cumulative numbers from the 50 siliques; the averages per plant and the corresponding statistical analysis are shown in Supplementary Fig. 2a. **c** Schematic representation and optical microscope images of transversal sections of pistils at different developmental stages in wild type, *bpc123*, *bpc4 bpc6*, and *bpcV*. Cell walls were stained with neutral red. Scale bar 20 μm

As BPCs were reported to be expressed throughout gynoecium development (Luna-García et al. 2024; Monfared et al. 2011), to better understand their role during fruit development, we focused our analyses on the following genotypes: wild type, *bpc1-2 bpc2 bpc3* (hereafter referred to as *bpc123*), *bpc4 bpc6* and *bpc1-2 bpc2 bpc3 bpc4 bpc6* (hereafter referred to as *bpcV*). While *bpc4 bpc6* siliques were found to be similar in length and seed-set to those of wild type plants, *bpc123* mutants exhibited short siliques with a reduced seed-set compared to wild type. Even though the mutation of BPCs of class II alone did not cause any visible defect, the *bpcV* mutants, in which both class I and class II BPCs are mutated, displayed an aggravation of the silique’s phenotypes, confirming the previously reported genetic interaction between BPCs of class I and II (Petrella et al. 2020) (Fig. 1A and Supplementary Fig. 3B-D).

Septum formation and morphology were evaluated in all the genetic backgrounds of interest, in green fully mature siliques. In wild type and *bpc4 bpc6* mutant, the septum is present and completely formed in 48/50 and 47/50 analyzed siliques, respectively, while the remaining siliques showed only partial septum formation. In *bpc123* only 12/50 siliques showed a completely developed septum, 36/50 exhibited a partial septum development and this structure was completely absent in 2/50 siliques. Septum developmental defects were exacerbated when mutants in BPC of class I and class II were combined. In *bpcV*, indeed, none of the analyzed siliques (0/50) were able to develop a complete septum and in 42/50 siliques the septum was absent (Fig. 1A-B and Supplementary Fig. 2).

These morphological analyses clearly showed that BPCs play a fundamental role during carpel development, in particular in septum specification and development. As the septum arises from the fusion of the two septum primordia, which differentiate within the CMMs along with the other medial tissues, between stages 8 and 10 of gynoecium development, *bpc4 bpc6*, *bpc123* and *bpcV* carpels have been studied along the course of these developmental stages (Fig. 1C).

In all the analyzed mutant backgrounds, at stage 8 and stage 9 CMMs start to enlarge and become visible and, at stage 10, in the wild type and *bpc4 bpc6*, the two septum primordia start to fuse giving rise to the septum whereas in *bpc123* and *bpcV* carpels at stage 10 this fusion does not happen, thus impairing septum development (Fig. 1A-B). This morphological defect becomes more evident at later developmental stages (stage 11/12) (Fig. 4E). These analyses strongly indicate that higher-order *bpc* mutants are more severely impaired in septum development, while BPCs of class II alone do not play a major role in this process. As so we decided to conduct further analyses focusing on *bpcV* and *bpc123.*

### BPCs are involved in the regulation of several septum developmental pathways

**Fig. 2:**
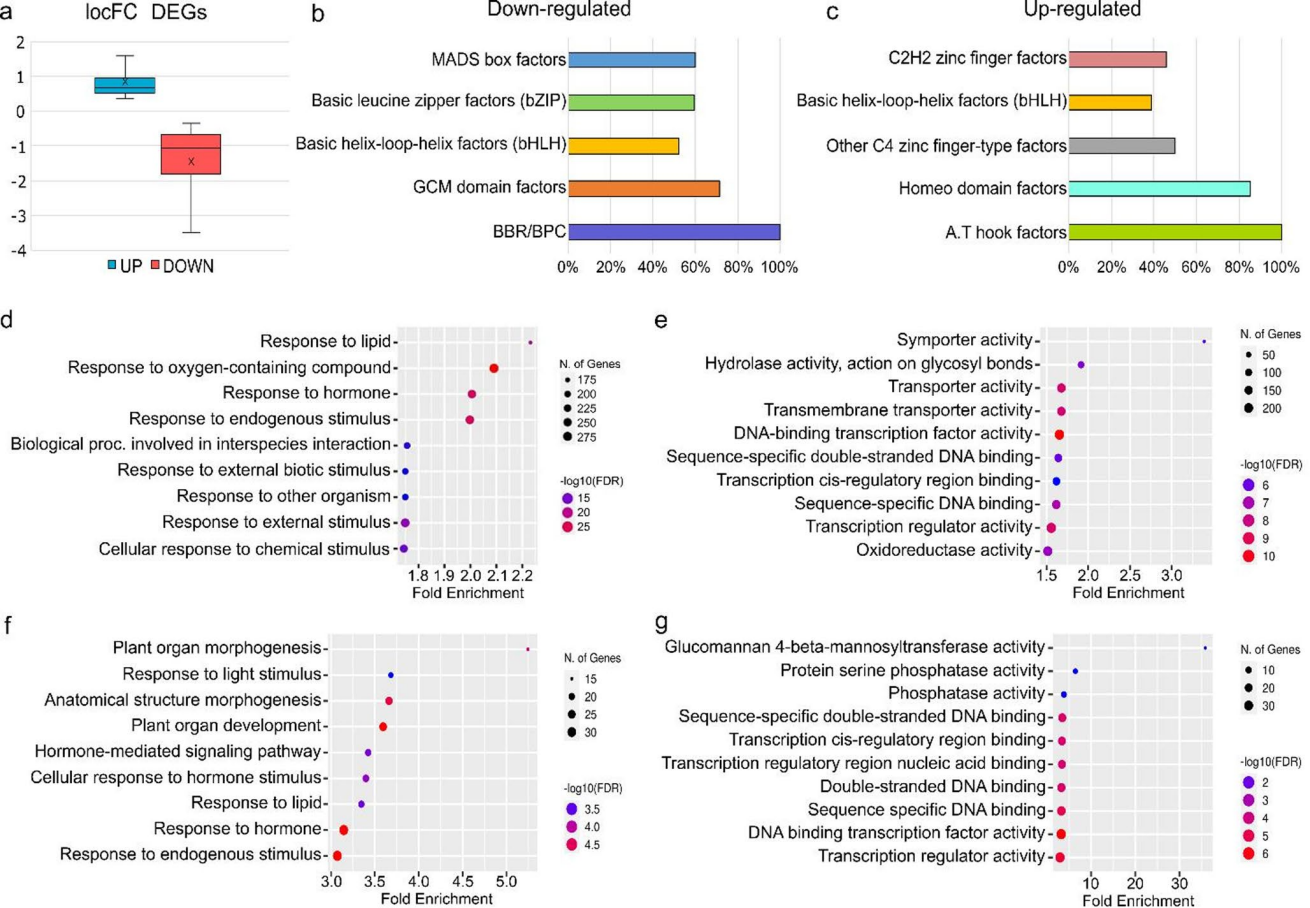
Differential gene expression analysis of wild type and *bpcV* RNAseq. **a** Boxplot showing the distribution of logFC of down-regulated and up-regulated DEGs. **b** and **c** TFBS enrichment analysis. Bars represent the proportion of TFs in TF family -as defined by Jaspar- showing a significant enrichment in the promoters of the Down-regulated and Up-regulated DEGs. **d** and **e** Gene ontology enrichment analysis of the 2253 DEGs for “biological processes” (BP) and “molecular function” (MF), the top 9 top 10 ontologies with the most significant enrichment are shown respectively for BP and MF GO analysis was performed using ShinyGo 0.76 with default parameters. **f** and **g** Gene ontology of intersected genes between DEGs and DAP-seq lists, divided into biological processes and molecular function

We aimed to identify target genes and molecular pathways directly or indirectly modulated by BPCs in gynoecium development. A differential gene expression analysis on inflorescence apices and flowers up to stage 12, which encompasses all the different steps of gynoecium development, was performed on wild type and *bpcV* samples. A total of 2253 genes were found differentially expressed and, among these, 1006 were up-regulated and 1247 down-regulated (Fig. 2A and Supplementary Table 1).

Functional enrichment showed that the 9 most enriched terms in the “biological process” domain of the gene ontology (GO, Fig. 2D and Supplementary Table 2) were associated with responses to endogenous and external stimuli, and hormones. Equivalent analyses recovered a highly significant enrichment in terms involved in transporter activity and DNA-binding activity when the “molecular function” domain of the gene ontology was considered (Fig. 2E and Supplementary Table 2).

Several transcription factors (TFs), known to participate in gynoecium and CMM development, as well as effector genes involved, for instance, in hormonal signaling and auxin transport, such as *PIN-FORMED7* (*PIN7*) and *PIN-LIKES3* (*PILS3*), were found to be deregulated in the dataset. Among the transcription factors, it is worth mentioning *SPATULA* (*SPT*) and *NO TRANSMITTING TRACT* (*NTT*), both downregulated, which play a major role respectively in the specification of CMM and transmitting tract (Crawford et al. 2007; Heisler et al. 2001).

To refine our understanding of the complex gene regulatory network controlling carpel development, an in-silico analysis of promoter sequences of the up- and down-regulated DEGs was performed to identify known transcription factor binding sites (TFBS) and associated TF families over-represented in our set of DEGs with the Pscan software (Zambelli et al. 2009). A total of 168 and 119 TFBS were found to be significantly enriched in the down- and up-regulated DEGs, respectively, suggesting that factors belonging to these families might be involved in carpel development, in parallel and/or downstream of the BPCs (Supplementary Table 3). Regulatory regions of the down-regulated DEGs displayed significant enrichment for G-box like motifs, bound by TF belonging to the bHLH and bZIP TF families (Ezer et al. 2017); C-boxes, associated with BPCs (Kooiker et al. 2005), W-boxes (WRKY genes-GCM domain factors (Ciolkowski et al. 2008) and then CArG-boxes, recognized by MADS domain factors (Muiño et al. 2014) (Fig. 2B).

Up-regulated DEGs displayed a significant enrichment of TFBS associated with the following families of TFs: A.T. hook motif, homeodomain factors, C2H2 zinc finger factors, other C4 zinc finger-type factors, and bHLH (Fig. 2C).

*A subset of transcription factors and effector genes are likely to be direct targets of BPCs*.

The binding profile of BPCs, as obtained by DNA Affinity Purification sequencing (DAP-seq) assay, was analyzed employing the free-access database Plant Cistrome (http://neomorph.salk.edu/PlantCistromeDB) (O’Malley et al. 2016). The database provides binding peaks for both BPC1 (representative of class I BPCs) and BPC6 (representative of BPCs of class II). A total of 175 DEGs had one or more peaks, obtained from DAP-seq, in their promoter (defined as 1000 upstream and 500 bases downstream to the annotated transcription start site TSS), which suggests a putative role of BPCs in their direct regulation (Supplementary Table 4).

A GO term functional enrichment analysis of these genes recovered a statistically significant enrichment in terms associated with plant organs and morphogenesis, response to hormones, and stimuli (Fig. 2F and Supplementary Table 5). The most over-represented “molecular function” GO terms were related to DNA-binding activity and transcription (Fig. 2G and Supplementary Table 5).

The genes associated with hormonal response and signaling include several hormone types, spanning from jasmonate to cytokinin and auxin, suggesting that the pleiotropic phenotype of the *bpcV* plants might be linked to the simultaneous alteration of different pathways. Furthermore, a consistent subset of these genes are enzymes directly involved in hormonal regulation rather than transcriptional regulation. In the auxin case, for instance, these putative direct BPCs targets appear to be involved in both its homeostasis (i.e. AT4G27260) and transport within the cell (i.e. *AUX1* and *PILS3*).

On the other hand, this putative direct targets list also contains transcriptional factors, already known to be specifically involved in gynoecium development in different conditions, such as TEOSINTE BRANCHED 1/CYCLOIDEA/PCF *4*,* NGATHA3*,* PHYTOCHROME INTERACTING FACTOR5* and *NO TRANSMITTING TRACT (*Ballester et al. 2021; Crawford et al. 2007; Reymond et al. 2012; Wang et al. 2024).

### BPCs of class I and II positively regulate SPATULA

**Fig. 3:**
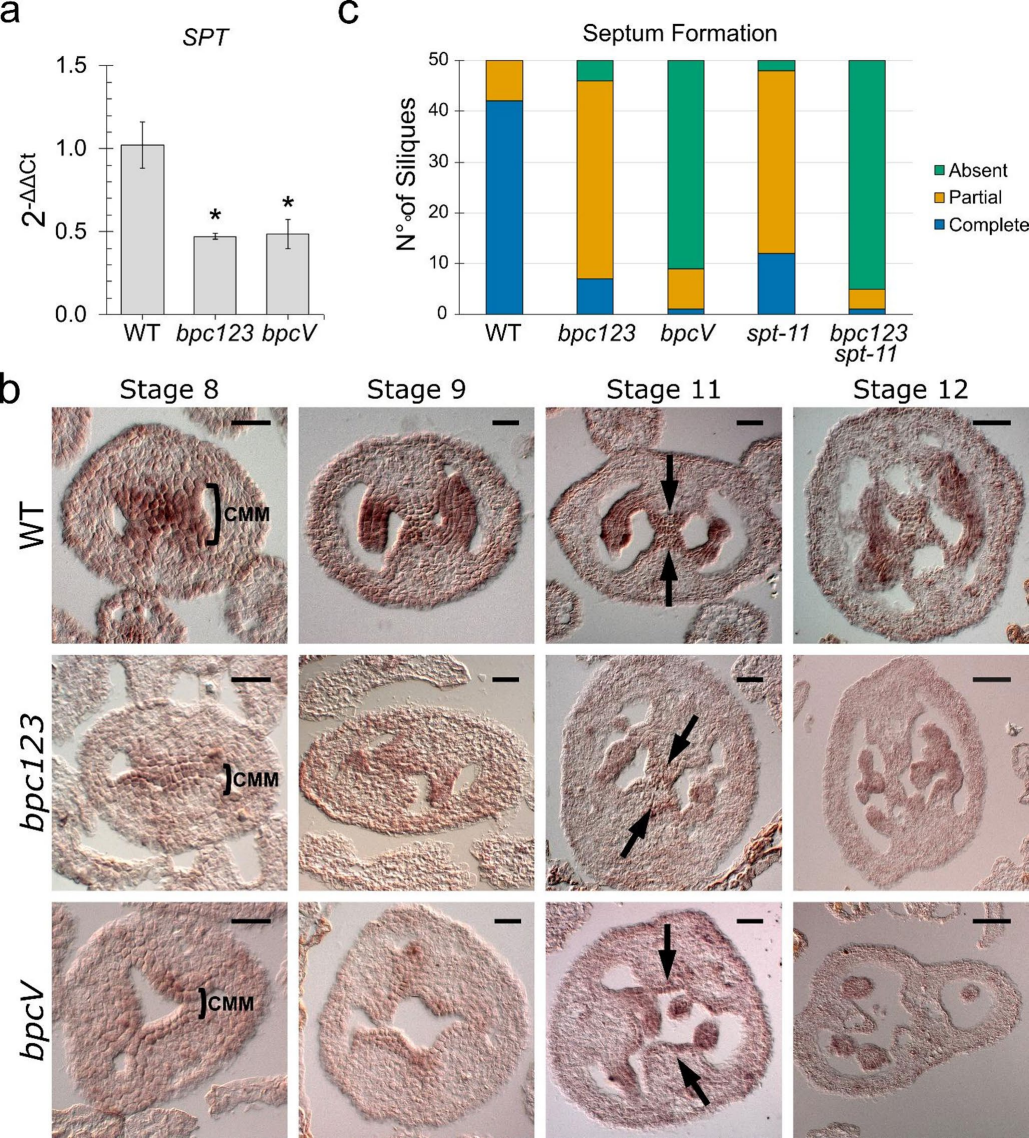
Relationship between *SPT* and *BPCs* **a** RT-PCR showing the expression of *SPT* in *bpc123* and *bpcV*. Expression of *SPT* was normalized to that of UBIQUITIN and the expression level in wild type was set to 1. The experiment was conducted on three biological replicates, and statistical significance was calculated using a t-test, * p value < 0.05. **b** In situ hybridization showing the expression profile of *SPT* in wild type, *bpc123*, and *bpcV* transversal carpel sections at different developmental stages. Brackets at stage 8 indicate the layers of CMM cells expressing *SPT.* Compared to the wild type the signal in *bpc123* and *bpcV* CMM appears to be weaker and confined in the outermost cell layers. At stage 11, the area between the two arrows includes the septum, which in the wild type is correctly fused and shows a strong *SPT* expression. In *bpc123* and *bpcV*, the expression of *SPT* in the same region is weaker and, in *bpcV*, the septum is not properly fused. Scale bar 20 μm (Stage 8, 9–10, 11) or 50 μm (Stage. 12). **c** Septum formation analyses considering a complete, partial or absent septum in wild type, *bpc123*, *bpcV*, *spt-11*, and *bpc123 spt-11*. 10 siliques were analyzed from 5 independent plants for a total of 50 siliques for each genotype. The chart displays the cumulative numbers from the 50 siliques; the averages per plant and the corresponding statistical analysis are shown in Supplementary Fig. 2b

Among the DEGs highlighted in our transcriptomic analyses, we focused on the bHLH transcription factor SPT, because it plays a fundamental role in gynoecium development, as it connects cytokinin and auxin signaling to the CMM and septum specification pathway (Reyes-Olalde et al. 2017). Moreover, *spt* mutants are characterized by a smaller gynoecium with severe defects in the septum and transmitting tract structures, unfused carpels in the apical part of the ovary, and smaller and wider siliques (Groszmann et al. 2008; Heisler et al. 2001; Reyes-Olalde et al. 2017). Not only *bpcV*, as already predicted by the RNAseq analysis, but also *bpc123* shows a downregulation of *SPT* expression in the whole inflorescence, suggesting that BPCs of class I play a prominent role in the regulation of *SPT*. (Fig. 3A).

To verify how the spatiotemporal expression profile of *SPT* is affected during CMM and septum differentiation, an in situ hybridization on transversal sections of flowers at different developmental stages was performed. In wild type carpels at stages 8 and 9, *SPT* is mainly expressed in the initiating and developing medial regions. At later developmental stages, the probe signal is detectable in the developing septum, transmitting tract, ovules, and funiculus (Fig. 3B). In both *bpc123* and *bpcV* carpels, *SPT* expression appears to be restricted to a reduced number of cell layers of the developing CMMs (stages 8 and 9) and septum (stages 11 and 12). Interestingly, these are the cells that in wild type plants should normally fuse, giving rise to the septum and transmitting tract (Fig. 3B).

To address the genetic relationship between BPCs and SPT, we analyzed the quadruple mutant *bpc123 spt-11*, in which the strong *spt-11* allele (Ichihashi et al. 2010) was introgressed in the *bpc123* background. The phenotypical defects of *bpc123 spt-11* closely resemble the ones of *bpcV;* plants are smaller compared to the wild type and show smaller siliques and a high number of non-fertilized ovules (Supplementary Fig. 3).

Only 12/50 *spt-11* siliques develop a complete septum while 21/50 siliques can only partially develop this structure. In 39/50 *bpc123* siliques, the septum is only partially developed. Interestingly, the *bpc123 spt-11* exhibits a more drastic phenotype than the triple and single *bpc123* and *spt-11*, respectively, showing the absence of septum in 45/50 siliques analyzed (Fig. 3C).

Taken together, these analyses helped in placing the BPCs upstream of *SPT* in the gene regulatory network leading to septum differentiation. Furthermore, the phenotypical characterization of the *spt-11* and *bpc123 spt-11* mutants hints that once activated, *SPT* controls a partially independent gene pathway that, in parallel to the one controlled by BPCs, contributes to septum development.

### Transmitting tract differentiation is directly regulated by BPCs

*bpc123* and *bpcV* siliques phenotype is characterized by a defective septum, caused by defects in septum fusion. The septum arises from the CMM, as the placenta, ovules, transmitting tract, stigma, and style, collectively known as the medial tissues.

The putative BPCs direct targets list includes the C2H2/C2HC zinc finger transcription factor *NO TRANSMITTING TRACT* (*NTT*), which was found to be downregulated in *bpcV* and to have a BPC-bound DAP-seq peak in its promoter. As the name suggests, the *ntt* mutant is severely impaired in transmitting tract differentiation, affecting pollen tube growth (Crawford et al. 2007; Herrera-Ubaldo et al. 2019).

**Fig. 4:**
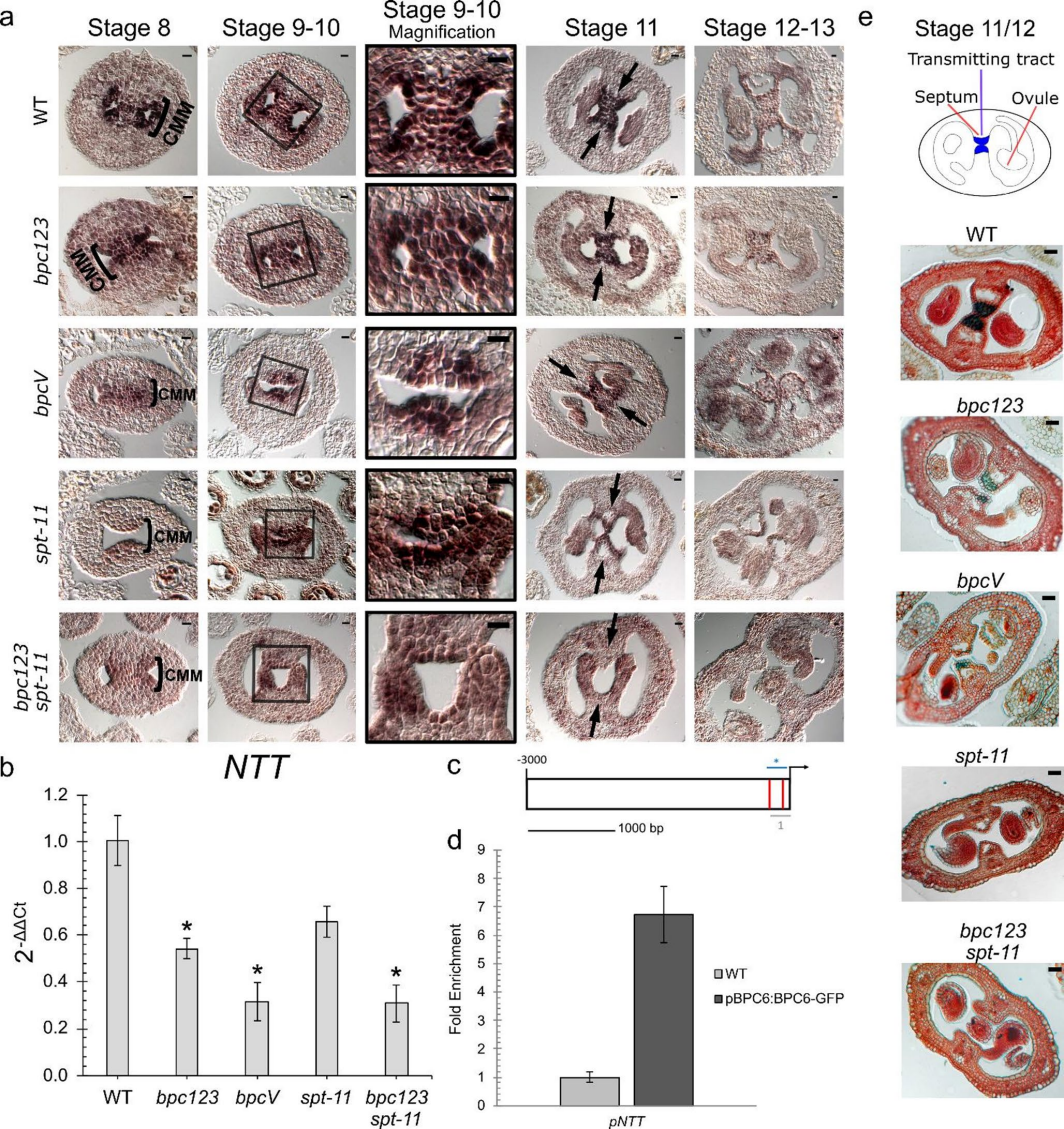
Transmitting tract differentiation is impaired in BPCs mutants **a** In situ hybridization evaluating *NTT* expression pattern in wild type, *bpc123*, *bpcV*,* spt-11* and *bpc123 spt-11* mutants in transversal carpel sections. Brackets at stage 8 indicate the layers of CMM cells expressing *NTT.* Compared to the wild type the signal in *the mutants* appears to be weaker and confined in only a few cell layers. The squared area at stage 9–10, which includes the CMM, is also shown magnified. At stage 11, the area between the two arrows includes the septum. Scale bar 20 (Stage 8, 9–10, 11) µm or 50 μm (Stage 12) **b** RT-PCR showing the expression of *NTT* in *bpc123*,* bpcV*,* spt-11* and *bpc123 spt-11*. Expression of *NTT* was normalized to that of UBIQUITIN and the expression level in wild type was set to 1. The experiment was conducted on three biological replicates, statistical significance was calculated using a t-test, *p value < 0.05. **c** Schematic representation of *NTT* promoter region from the start codon to -3000 bp. Red lines represent C-boxes; grey line represents the selected region for ChIP assay; blue line and asterisk represent DAP-seq peak coordinates. **d** ChIP results of one representative experiment out of three independent biological replicas showing fold enrichment values of region 1 of *NTT* promoter in the *pBPC6:GFP-BPC6* line compared to wild-type. **e** Schematic representation of a transversal gynoecium at stage 11–12, Transversal sections of gynoecium at stage 11–12, stained with neutral red to visualize the cell wall and alcian blue to visualize the extracellular matrix of the transmitting tract. Scale bar 50 μm

In wild type pistils, at stages 8 and 9, *NTT* is expressed in the medial domain, where the two septum primordia are specified. At later stages, its expression marks the medial tissues and the developing septum and transmitting tract. While in *bpc123* this pattern seems to be maintained, *bpcV* shows a reduction in the *NTT* probe signal, particularly in stages 8–10 supporting the RNAseq results. Indeed, comparing the expression pattern of *NTT* in the wild type and *bpcV* starting at stages 9 and 10, it is possible to observe a reduction in the number of CMM cells in which this gene is expressed (Fig. 4A). The different downregulation levels of *NTT* in *bpc123* and *bpcV* were further confirmed via RT-PCR and correlate well with the different severity of phenotypes registered in the two mutants (Fig. 4B).

To better understand the gene regulatory network controlled by BPCs and the role the SPT plays in it, we decided to investigate the expression pattern of *NTT* in *spt-11* and *bpc123 spt-11.* From stages 9 and 10, similarly to what was observed in *bpcV*, the *NTT* signal is confined in fewer cells in *spt-11* compared to the wild type. This reduction appears even more drastic in the *bpc123 spt-11* quadruple mutant, suggesting that the BPCs and SPT can autonomously control *NTT* expression and therefore, probably, septum development and fusion (Fig. 4A-B).

Our analysis of publicly available DAPseq data suggested that BPCs can directly bind the *NTT* promoter on a region close to the translational start site characterized by the presence of two C-boxes, the BPCs binding motif (Fig. 4B). To confirm the binding in vivo in the tissues of interest, a Chromatin Immunoprecipitation (ChIP) assay was carried out employing the *pBPC6:GFP-BPC6* line. The *NTT* promoter region corresponding to the DAPseq coordinate was found to be highly enriched in this line, further supporting the idea that *NTT* expression is directly induced by BPCs (Fig. 4C).

We next wondered whether *NTT* downregulation is correlated with transmitting tract defects in the BPC mutants. In wild type carpels at stage 12 this tissue is completely differentiated and the extracellular polysaccharidic matrix can be stained with alcian blue. In *bpc123* and *bpcV* carpels, instead, only a few cells display a weak blue color, indicating an impairment in transmitting tract specification. Interestingly, the ability to differentiate the transmitting tract is also lost in *spt-11* and *bpc123 spt-11* quadruple mutants, confirming a genetic interaction between this gene and the BPCs (Fig. 4D-E).

### BPCs control PILS3 expression within the septum

The correct patterning and differentiation of the CMM partially relies on precise auxin distribution, which is in some measure controlled by SPT (Reyes-Olalde et al. 2017). Interestingly, the DEG list in the *bpcV* mutant was significantly enriched in terms related to hormonal response and transporter activities (Fig. 2B-C). Among the genes sharing this function, *PILS3* was found to be moderately downregulated (LogFC − 0.55, FDR 0.015) and its promoter regions contain a BPC-bound DAPseq peak, making it a potential direct target of BPCs. PILS proteins are intracellular auxin transport facilitators at the endoplasmic reticulum, where they control cytoplasmic availability of auxin, contributing in this way to various growth processes (Barbez et al. 2012; Béziat et al. 2017; Feraru et al. 2019; Waidmann et al. 2023).

**Fig. 5:**
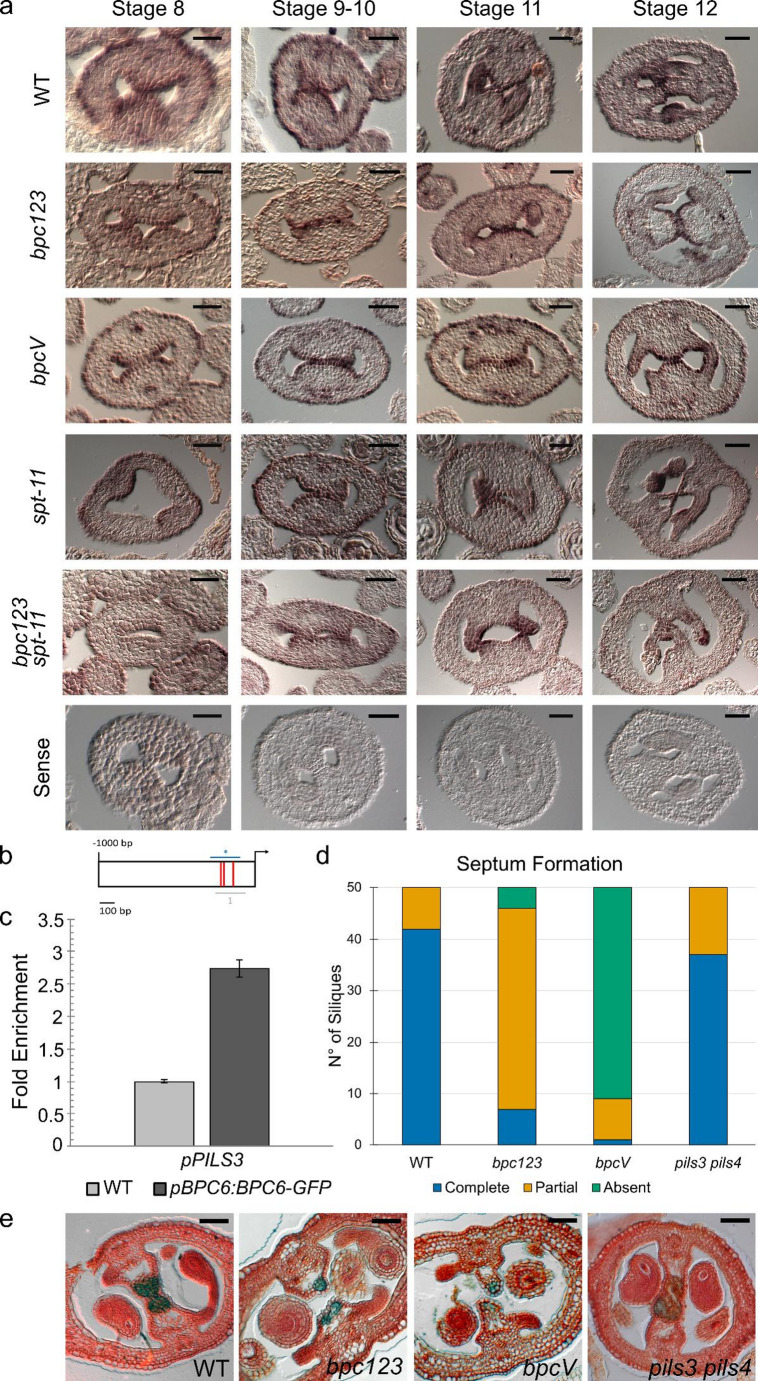
PILS3 involvement in carpel medial region differentiation **a** In situ hybridization evaluating *PILS3* expression pattern in wild type, *bpc123*, *bpcV*,* spt-11* and *bpc123 spt-11* mutants in transversal carpel sections. To validate the specificity of the newly designed *PILS3* probe, the same tissues employed for the expression pattern evaluation were also hybridized with the sense probe, which gave no visible signal within the carpels. Scale bar 20 (Stage 8, 9–10, 11) µm or 50 μm (Stage 12). **b** Schematic representation of *PILS3* promoter region from start codon to -3000 bp. Red lines represent C-boxes; grey line represents the selected region for ChIP assay; blue line and asterisk represent DAP-seq peak coordinates. **c** ChIP results of one representative experiment out of three independent biological replicas showing fold enrichment of *pBPC6:GFP-BPC6* line compared to wild-type in *PILS3* promoter. **d** Septum formation analysis in wild type, *bpc123*, *bpcV*, and *pils3 pils4* mutants. 50 siliques were considered for each genotype. To minimize any potential fluctuations in the controls, *pils3 pils4*, and *spt-11* siliques (Fig. 2B) were analyzed in the same experiments. Therefore, the data displayed here for the wild type, *bpc123* and *bpcV* are the same as the ones in Fig. 2B. The chart displays the cumulative numbers from the 50 siliques; the averages per plant and the corresponding statistical analysis are shown in Supplementary Fig. 2b. **e** Alcian blue and neutral red staining of wild type, *bpc123*,* bpcV* and *pils3 pils4* carpels at stages 11–12, scale bar 50 μm

The expression pattern of *PILS3* was investigated via in situ hybridization in wild type pistils at different developmental stages, to start to elucidate the possible role of this transporter within the septum. Interestingly, the expression of this transporter at stages 8–10 was found to be confined in the outermost cell layer of the CMM, which is the one responsible for septum fusion, and in the external epidermal layer of the carpel. At later stages, *PILS3* is also expressed in the funiculus and developing ovules. The pattern of expression of *PILS3* is conserved in the *bpc123*,* bpcV*, and *spt-11* mutants. In *bpc123 spt-11* the signal seems to be more diffused, probably because when both *BPCs* of class I and *SPT* are mutated, septum formation is severely affected (Fig. 5A). Consistent with the in situ hybridization results, *PILS3* mild deregulation was not clearly confirmed by RT-PCR, probably also due to the low level of expression of this gene.

The DAPseq analysis revealed the presence of a sequence in the promoter of *PILS3*, containing three C-boxes, likely bound by BPCs (Fig. 5B). The binding of BPCs to this region was confirmed in vivo in Arabidopsis inflorescences through ChIP, further corroborating the hypothesis that *PILS3* is a direct target of BPCs during gynoecium development (Fig. 5C).

The PILS family is composed of 7 members, with *PILS3* and *PILS4* located in tandem in the genome, likely originating from a duplication event. The sequence homology between *PILS3* and *PILS4* and their similar expression pattern strongly suggest that these genes might act redundantly (Barbez et al. 2012). We hence used CRISPR/CAS9 gene editing technology to establish a *pils3 pils4* loss of function double mutant, allowing us to overcome potential redundancy during pistil development. Macroscopically, *pils3 pils4* siliques appear to be wild type-like, with 38/50 siliques able to develop a complete septum (Fig. 5D). However, alcian blue staining on transversal carpel sections revealed a reduction in the extracellular matrix secreted by the transmitting tract cells, suggesting that a fine-tuned control of auxin availability within the septum cells compartments is fundamental for the proper specification of the medial domain components (Fig. 5E).

## Discussion

BPCs transcription factors act as activators and repressors of their targets and are likely to be redundantly involved in regulating several different steps of Arabidopsis development. A good indication of their fundamental role comes from the observation that the simultaneous mutation of combinations of these genes causes the onset of several phenotypical defects (Monfared et al. 2011; Simonini et al. 2014; Petrella et al. 2020).

Here we enlighten the role of BPCs in gynoecium development. It is not always straightforward to determine whether differences in gene expression across different mutant backgrounds are due to the mutation itself or are the result of the observed phenotypical alterations. Here we employed a combination of molecular and genetic tools to show that *bpcV* and, to a lesser extent *bpc123*, are impaired in septum formation and that this impairment can be at least partially linked to the gene regulatory network controlled by these transcription factors.

In Arabidopsis, the septum is the structure that divides the two carpels. It contains the transmitting tract, and differentiates from the CMM, along with other fundamental structures of the so-called medial domain, such as ovules and placenta. *SPT* is expressed in the CMM and it is required for the proper specification of the medial domain tissues. Hence, *spt* mutants are characterized by a reduced seed set and short siliques with a defective septum, which does not fuse properly (Alvarez and Smyth 1999; Heisler et al. 2001).

To further elucidate the genetic relationship between *SPT* and the *BPCs*, we generated the *bpc123 spt-11* mutant. *bpc123* and *spt-11* show similar septum defects, where the majority of their siliques show a defective or only partially fused septum. Interestingly, knocking down *SPT* in *bpc123* causes the complete loss of septum in almost all the analyzed siliques, suggesting there is an additive relationship between these genes.

It is indeed possible that SPT and the BPCs of class I control two partially independent pathways and that the simultaneous mutation of *BPCs* of class I and *SPT* leads to the exacerbation of the septum defects in *bpc123 spt-11* compared to the two parental lines.

Besides *SPT*, it is likely that there are other factors involved in septum specification directly or indirectly regulated by the BPCs. The RNAseq experiment on both wild type and *bpcV* plants helped to identify these factors. Indeed, we identified 1106 up and 1247 down-regulated genes, involved both in gene regulation and biological processes, such as transmembrane transport.

It is worth mentioning that two TFs of the *PHYTOCHROME INTERACTING FACTORs* family, *PIF4* and *PIF5* were also included in the downregulated genes list. These proteins participate in the activation of the shade avoidance response and, in shaded conditions, can act in parallel to *SPT* to promote carpel margin differentiation (Reymond et al. 2012). Furthermore, the enrichment of GO terms related to hormonal response and transmembrane transport activity suggests that the BPCs might also directly regulate the hormonal signaling cascade and transport throughout the inflorescence.

To gather more information on the gene regulatory network involving the BPCs, a TFBS enrichment analysis was carried out on the DEGs’ promoter regions.

In addition to C-boxes, bound by BPCs, we found an enrichment of motifs associated with the bZIP and bHLH TF families, which are known to regulate processes including light and stress signaling, seed maturation, and flower development (Hao et al. 2021). Since *SPT* is a member of the bHLH family, it is possible to speculate that some of the deregulated genes in our dataset, which contain bHLH binding sites in their promoters, could be direct targets of SPT, and that their misregulation might result from *SPT* downregulation in *bpcV* mutant.

CArG boxes, normally bound by MADS domain transcription factors, were also found among the enriched motifs identified in the dataset. This family contains well-characterized homeotic genes, responsible for the specification of the different floral organs, and several pivotal regulators of inflorescence development (Alvarez-Buylla et al. 2010). Genes regulated by the MADS domain factors are thus likely to be involved in the developmental patterning of the inflorescence and floral meristems. The presence of many CArG boxes in the promoters of the DEGs list suggests that these pathways might be directly and indirectly regulated also by the BPCs, and that some of the phenotypical defects of the *bpcV* might be linked to the deregulation of MADS targets. It is also worth mentioning that BPCs of class I and II interact with the MADS domain protein SVP, forming a complex that regulates the expression of the homeotic gene *STK* (Petrella et al. 2020; Simonini et al. 2012). The enrichment of CArG boxes containing promoters in the DEG list hints at the existence of general mechanisms where BPCs-MADS complexes can cooperatively regulate the expression of a set of genes, which is crucial for the development of reproductive structures.

**Fig. 6:**
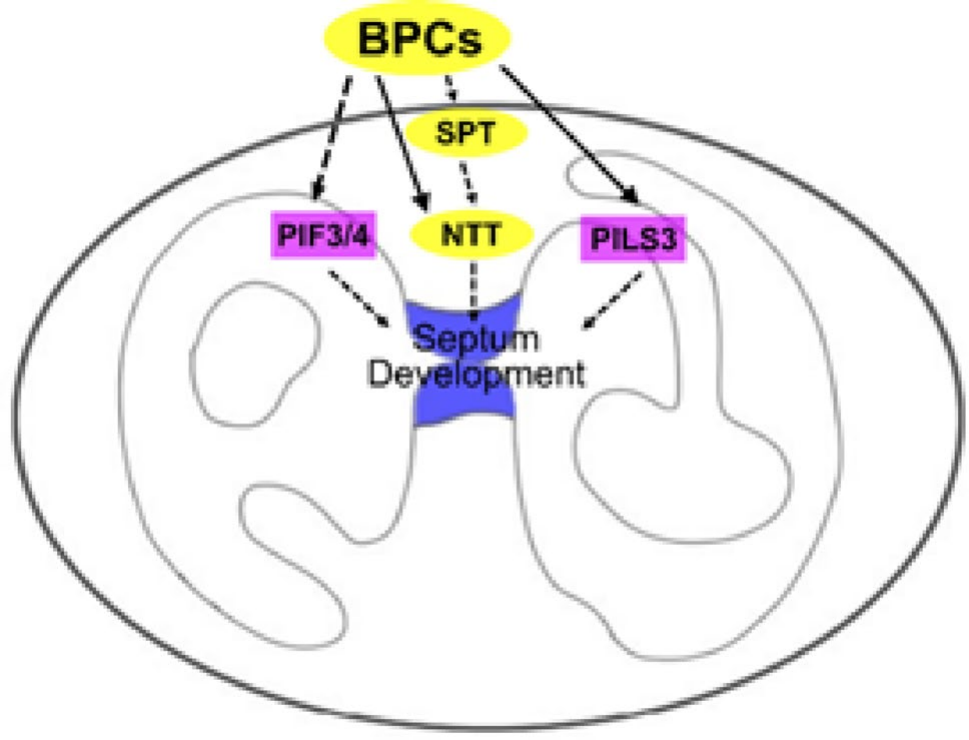
Working model. BPCs of class I and class II serve as key regulators of septum development by orchestrating a complex gene regulatory network. This network includes both transcriptional regulators (*SPT*,* NTT –* shown as yellow ellipses in the model) and effector proteins (such as *PILS3*,* PIF3*, and *PIF4* – shown as magenta squares in the model). In particular, BPCs indirectly regulate the expression of *SPT*, a master regulator of gynoecium development, and directly regulate both *NTT* and *PILS3*. SPT can act in parallel with the BPCs to control the expression of *NTT.* Direct regulations are shown as black arrows, indirect regulations as dashed arrows

From the intersection of the DEG list with the putative BPCs binding sites in the Arabidopsis genome, we identified 175 putative direct targets of BPCs. Notably, the list generated by this analysis supports the model proposed above, where BPCs may play a central role in a gene regulatory cascade, controlling both the expression of TFs and effectors and orchestrating several developmental processes within the gynoecium (Fig. 6).

For instance, two members of the TEOSINTE BRANCHED 1/CYCLOIDEA/PCF family, *TCP4* and *TCP10*, were downregulated in our dataset. TCP4, together with other members of its family, controls the apical patterning of the gynoecium and can directly control the expression of *NGATHA3* (*NGA3*), which is also downregulated in *bpcV.* NGA3 is in turn involved, together with SPT, in stigma determination. One of the roles of TCPs, NGA3, and SPT is the control of auxin biosynthesis and transport (Ballester et al. 2021; Wang et al. 2024). Interestingly, since the majority of the genes involved in this regulatory network were found in the putative BPCs direct targets list, it is possible to speculate that the BPCs play an important role in gynoecium development during stigma determination.

One other gene with an important well-known function in gynoecium development is *NTT*, which is a master regulator of transmitting tract differentiation. Besides being present in the list of the BPCs putative direct targets, we demonstrated via in situ hybridization that *NTT* expression is specifically reduced in the CMM and septum in *bpc* multiple mutant combinations and that its promoter is directly bound by BPC6. The extracellular polysaccharidic matrix secreted by the transmitting tract cells is strongly reduced in *bpc123* and completely absent in *bpcV*, suggesting that the BPCs sit on top of the gene-regulatory cascade which leads to the determination of the transmitting tract. We notably demonstrated that *spt-11* mutants also show defects in the transmitting tract development. Notably, several ABCG transporters involved, among other things, in extracellular matrix deposition in reproductive organs were found to be downregulated in our RNAseq dataset (Herrera-Ubaldo et al. 2019; Panikashvili et al. 2010).

This observation gives strength to the hypothesis that BPC of class I and II orchestrate septum development by controlling the expression of master regulators (*SPT*) and directly triggering specific processes (i.e. *NTT* and transmitting tract differentiation). Furthermore, it also hints that the list of putative direct targets generated in this study can be a valid starting point to try to elucidate the causes of some of the pleiotropic inflorescence defects displayed by the *bpcV* mutant.

CMM differentiation and development are tightly linked to the action of auxin and cytokinin. Their asymmetric distribution among the cells of the gynoecium ensures the correct patterning of the tissues of the medial domain (Reviewed in Reyes-Olalde and de Folter 2019). Auxin, in particular, is synthesized in the CMM and then polarly accumulates into the lateral domains of the gynoecium (Reyes-Olalde et al. 2017). Auxin gradients across plant tissues are mainly generated by its directional intercellular transport and by its intracellular compartmentalization. This second mechanism indeed determines the amount of auxin within the cell which can enter the nucleus and trigger the auxin signaling response, and it is in part controlled by the PILS transporters. These proteins localize on the endoplasmic reticulum membrane and presumably extrude auxin into the ER lumen, sequestering it from the cytosol (Barbez et al. 2012; Feraru et al. 2019). Despite being mildly downregulated in *bpcV*, the expression profile of *PILS3*, which has been characterized for the first time in reproductive development here, suggests that it could be a good candidate player during septum development, as it appears to be expressed in the outermost cell layers of the CMM. Furthermore, *PILS3* appears to be a direct target of BPC6. Notably, the mutation of *PILS3* and its homolog *PILS4* causes the insurgence of problems in septum fusion, at low penetrance. Also, in this mutant, analyzing the extracellular matrix accumulation with alcian blue, the transmitting tract is not as developed as in the wild type, similar to *bpcV* and *spt-11*.

GO analysis of the DEG revealed significant enrichment in terms related to hormone responses and transporter activity. This suggests that BPCs contribute to the fine-tuning of these complex processes during septum development by finely regulating a broad range of factors, rather than causing major shifts in the expression of a few key genes. The simultaneous deregulation of other factors, besides *PILS3*, might explain the more drastic phenotype observed in the *bpc* mutants compared to *pils3 pils4.*

Because of their crucial roles and the fact that they belong to a relatively small family, the BPCs are interesting candidates to be studied in order to broaden our understanding of plant development. Here, for instance, through the characterization of septum development in *bpcV*, we were able to link together already known regulators of this process, such as *SPT* and *NTT*, and novel candidates, such as *PILS3.*

## Supplementary Information

Below is the link to the electronic supplementary material.

Supplementary Material 1. Supplementary Table 1: List of differentially expressed genes.

Supplementary Material 2. Supplementary Table 2: Best 10 enrichment GO terms of DEGs.

Supplementary Material 3. Supplementary Table 3: TFBS and associated TF families over-represented in our set of DEGs.

Supplementary Material 4. Supplementary Table 4: Intersection between DEG and DAP-seq data for BPC1 and BPC6.

Supplementary Material 5. Supplementary Table 5: Best 10 enrichment GO terms of DEGs U DAP.

Supplementary Material 6. Supplementary Table 6: List of primers employed.

Supplementary Material 7. Supplementary Fig. 1: *pils3* and *pils4* CRISPR/Cas9 genome edited mutants. Supplementary Fig. 2: Septum morphology analysis per plant. Supplementary Fig. 3: *bpc123 spt-11* phenotypical characterization.

## Data Availability

The datasets generated during and analysed during the current study are available in the GEO repository, https://www.ncbi.nlm.nih.gov/geo/query/acc.cgi?acc=GSE301158. Accession GEO: GSE301158, token for review: sdulowuwxhkfhmd.
