# Supplementary material for: A novel role of BPCs in the control of medial domain differentiation during gynoecium development in Arabidopsis thaliana
Source: Plant Mol Biol. 2025 Dec 23;116(1):4. doi: 10.1007/s11103-025-01662-x (PMC12727758; doi:10.1007/s11103-025-01662-x)
Supplement: Supplementary file 7 — Supplementary Material 7. Supplementary Fig. 1: pils3 and pils4 CRISPR/Cas9 genome edited mutants. Supplementary Fig. 2: Septum morphology analysis per plant. Supplementary Fig. 3: bpc123 spt-11 phenotypical characterization. [file 11103_2025_1662_MOESM7_ESM.docx]

**A novel role of BPCs in the control of medial domain differentiation**

**during gynoecium development in *Arabidopsis thaliana***

Francesca Caselli^1*^ Micaela Palermiti ^1*^, Rosanna Petrella^1^; Veronica Astrid Morlacchi^1^, Kai Dünser^2,3,4^,

Jűrgen Kleine-Vehn^2,3,4^, Matteo Chiara^1^, Veronica Gregis^1^

^1^Dipartimento di Bioscienze, Università degli Studi di Milano, Italy

^2^Institute of Biology II, Chair of Molecular Plant Physiology (MoPP), University of Freiburg, 79104 Freiburg, Germany

^3^Center for Integrative Biological Signalling Studies (CIBSS), University of Freiburg, 79104 Freiburg, Germany

^4^Institute of Molecular Plant Biology (IMPB), University of Natural Resources and Life Sciences (BOKU), Vienna, 1190 Vienna, Austria

*These authors contribute equally to this work

Corresponding author: veronica.gregis@unimi.it

**Supplementary Materials**

**Supplementary Table 1.** List of differentially expressed genes.

**Supplementary Table 2.** Best 10 enrichment GO terms of DEGs.

**Supplementary Table 3.** TFBS and associated TF families over-represented in our set of DEGs.

**Supplementary Table 4.** Intersection between DEG and DAP-seq data for BPC1 and BPC6.

**Supplementary Table 5.** Best 10 enrichment GO terms of DEGs U DAP.

**Supplementary Table 6.** List of primers employed.

**Supplementary Figure 1*.*** *pils3* and *pils4* CRISPR/Cas9 genome edited mutants.

**Supplementary Figure 2.** Septum morphology analysis per plant

**Supplementary Figure 3*.*** *bpc123 spt-11* phenotypical characterization.

**
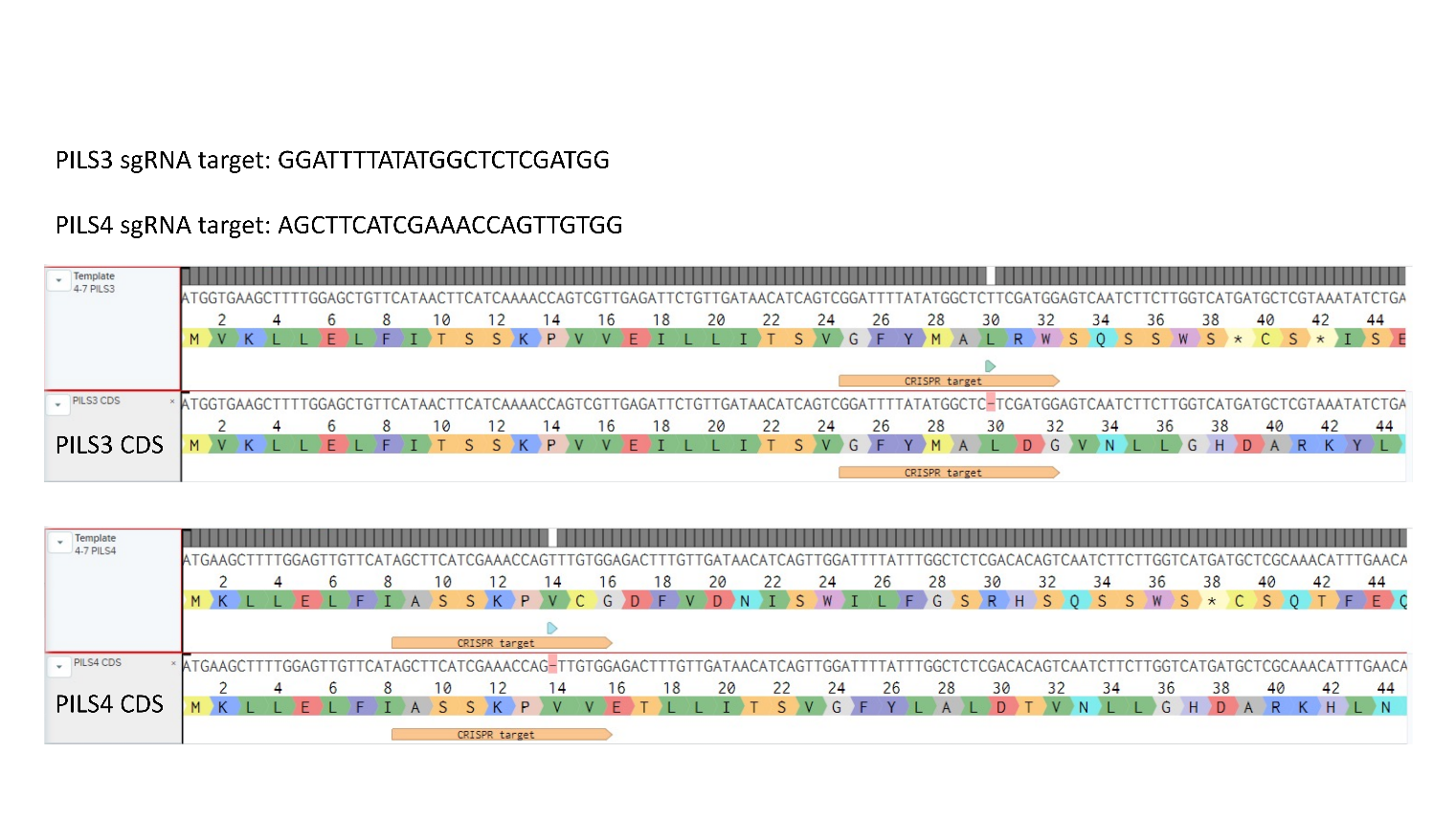
Supplementary Figure 1*.*** *pils3* and *pils4* CRISPR/Cas9 genome edited mutants. For each gene, the comparison between the mutated (top) and wild type (bottom) CDS is shown. In both cases, in the protospacer region it is possible to observe a 1 bp deletion, which leads to the formation of a premature stop codon.

**
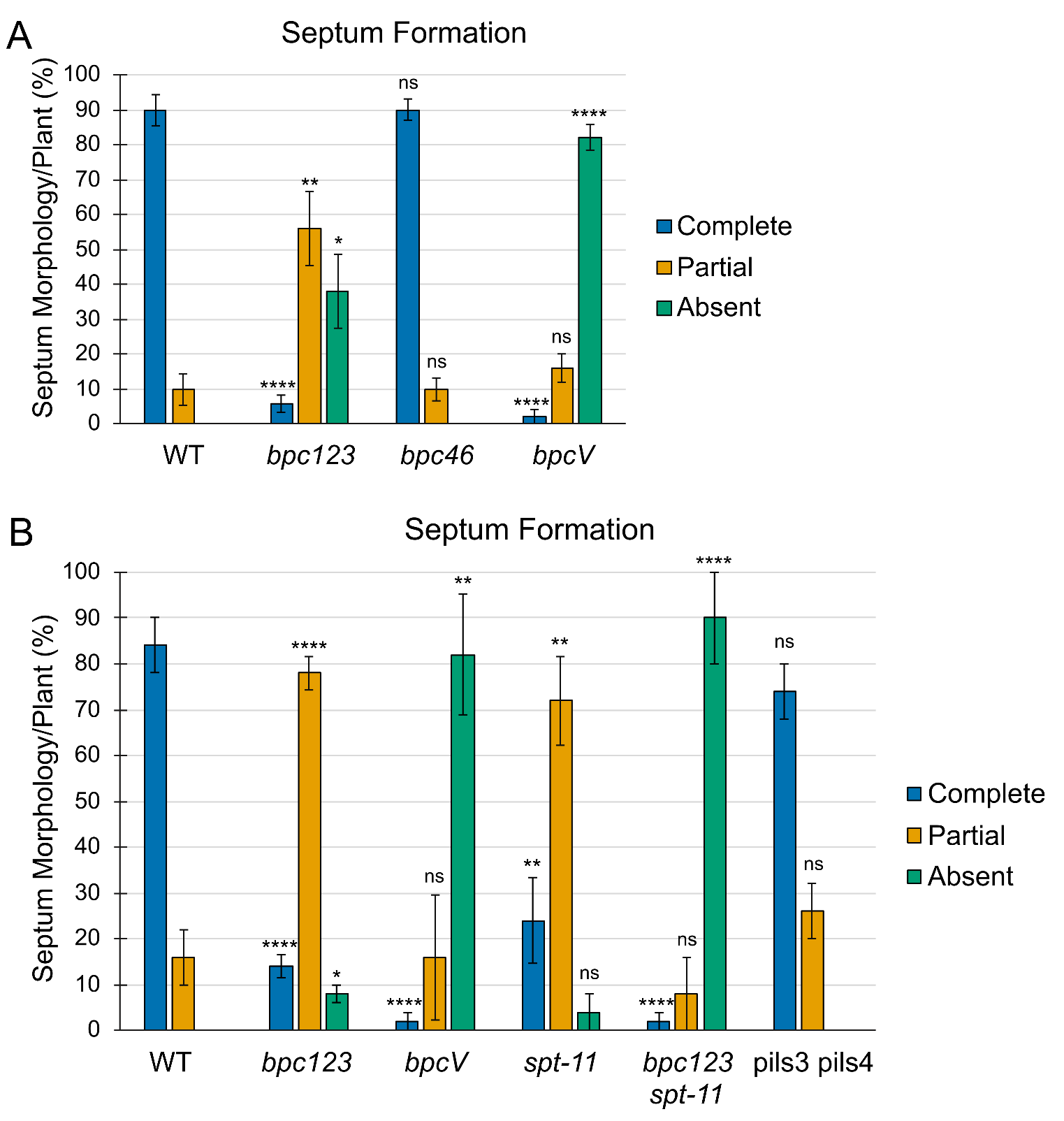
Supplementary Figure 2.** Septum morphology analysis. For each genotype, 10 siliques were analyzed from each of 5 individual plants (n = 50 siliques per genotype). The graph shows the average percentage of siliques with a complete/partial/absent septum per plant. Statistical significance between wild type and the different mutants, for each morphological category, was calculated with a t-test (ns not significant; * p value < 0.05; ** p value < 0.01; **** p value < 0.00001).


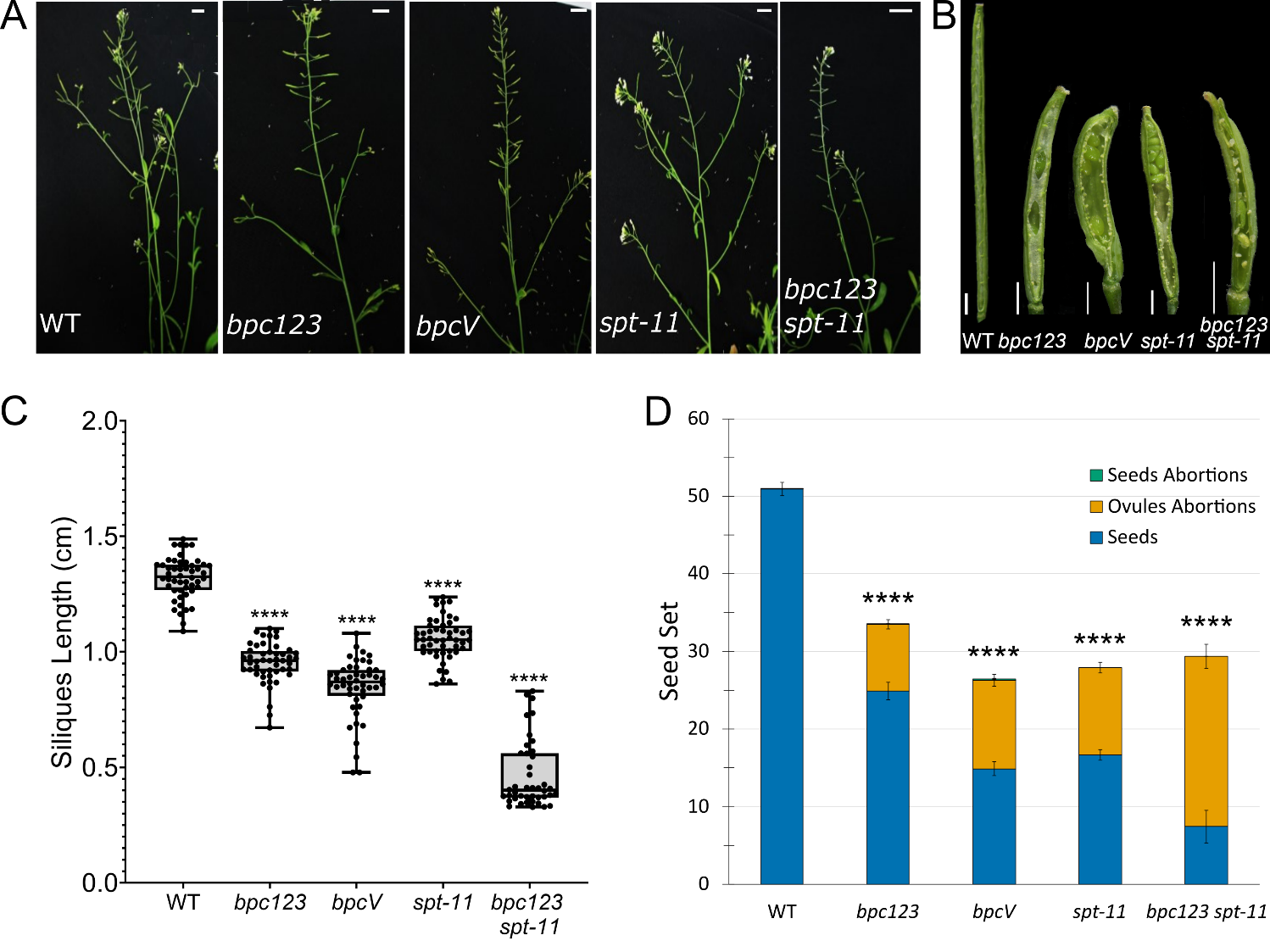
**Supplementary Figure 3*.*** *bpc123 spt-11* phenotypical characterization **(A)** Photo of the inflorescence of wild type, *bpc123, bpcV, spt-11* and *bpc123 spt-11* mutants. Scale bar 1 cm. **(B)** representative photo of a silique and its seed set of wild type, *bpc123, bpcV, spt-11* and *bpc123 spt-11* mutants. Scale bar 1 mm. **(C)** Siliques length differences between wild type and *bpc123, bpcV, spt-11* and *bpc123 spt-11* mutants. **(D)** Seed set analysis in wild type, *bpc123, bpcV, spt-11* and *bpc123 spt-11* mutants. The number of seeds, ovules and seeds abortions and the length of the siliques were determined for 50 siliques (10 siliques from 5 independent plants for each genotype). The statistical significance was calculated using an Anova test followed by Tukey HSD test (**** p < 0.0001). The siliques were analyzed when the inflorescence meristems were completely consumed.
